# Gene set variation analysis-based aging and senescence score as a prognostic indicator and therapeutic guide in lung adenocarcinoma

**DOI:** 10.3389/fgene.2023.1176292

**Published:** 2023-07-04

**Authors:** Tao Lin, Hong Wang, Ying Liu, Fangchao Zhao, Xi He

**Affiliations:** ^1^Department of Thoracic Surgery, Tangshan People’s Hospital, Tangshan, China; ^2^ Tangshan Central Blood Station, Tangshan, China; ^3^Department of Neurology, Xingtai Third Hospital, Xingtai, China; ^4^Department of Thoracic Surgery, The Second Hospital of Hebei Medical University, Shijiazhuang, China; ^5^Department of Thoracic Surgery, Dongguan Marina Bay Central Hospital, Dongguan, China

**Keywords:** aging, senescence, lung adenocarcinoma, prognosis, immunotherapy, tumor microenvironment

## Abstract

Accumulating evidence suggests that aging and senescence play crucial roles in tumorigenesis, cancer progression, and treatment. However, the influence of aging and senescence-related genes (ASRGs) on clinical outcomes and treatment options in lung adenocarcinoma (LUAD) patients remains unknown. Here, we developed an aging and senescence-related scoring system, ASRS, by integrating bulk transcriptome data from 22 LUAD datasets. In 3,243 LUAD samples, higher ASRS scores were associated with poor tumor stage and pathological grade, as well as shorter overall survival, disease-free survival, and recurrence-free survival. Additionally, ASRS was associated with different immune patterns in the tumor microenvironment (TME). Importantly, ASRS was found to predict therapeutic efficacy, with patients having a low ASRS benefiting from immunotherapy and those with a high ASRS responding better to chemotherapy. Therefore, ASRS represents a previously overlooked characteristic of LUAD that can influence patient outcomes and treatment success.

## Introduction

Lung cancer is the second most prevalent malignancy worldwide and is responsible for the majority of cancer-related deaths due to its rapid progression and distant metastasis (Sung et al., 2021). In China, lung cancer accounts for 37.0% and 39.8% of the world’s incidence and mortality, respectively, with a 5-year survival rate of only 10%–15% (Thai et al., 2021). Among all pathological types of lung cancer, lung adenocarcinoma (LUAD) currently predominates. Despite recent advances in comprehensive treatments, metastasis remains a significant obstacle to achieving favorable clinical outcomes (Wu et al., 2021). Various therapeutic modalities, particularly immunotherapy, have recently emerged as crucial components of therapy and have demonstrated potent protective effects on LUAD patients (Brody, 2020). However, there are substantial differences in drug response even among patients with similar clinicopathological characteristics (Singh et al., 2021). This suggests that conventional classifications, particularly the pathological TNM staging system, are inadequate for accurately predicting therapeutic response. Consequently, there is an urgent need to develop a novel molecular feature that can precisely identify subgroups of LUAD patients who are more likely to benefit from therapeutic regimens.

Cellular senescence, a state of irreversible cell cycle arrest, has long been regarded as an antitumor mechanism. This phenomenon was first observed in the early 1960s by Hayflick and Moorhead in their classical experiments, where human diploid fibroblasts cultured *in vitro* ceased to proliferate after a certain number of divisions. It was speculated that cellular senescence may play an essential role in suppressing tumorigenesis, as opposed to the indefinite proliferation exhibited by cultured tumor cells (Hayflick and Moorhead, 1961). Subsequent studies showed that overexpression of several oncogenes, such as RAS signaling pathway genes, RAF, MEK, MOS, and BRAF, can induce cellular senescence (Serrano et al., 1997; Campisi, 2005; Braig and Schmitt, 2006; Mooi and Peeper, 2006). Moreover, many experiments have highlighted the significance of cellular senescence as an important antitumor defense mechanism and a safe control mechanism to prevent tumor transformation of cells in the organism (Prieur and Peeper, 2008; Chandeck and Mooi, 2010). Recently, the use of aging-related gene signatures as diagnostic and prognostic biomarkers has attracted considerable attention from cancer researchers. Nevertheless, the molecular mechanisms and predictive functions of aging and senescence-related genes (ASRGs) in LUAD remain to be elucidated.

In this study, we analyzed the GSVA enrichment of ASRGs in 22 independent LUAD cohorts and uncovered the involvement of the aging and senescence-related score (ASRS) in LUAD progression, immune cell infiltration, immunomodulators, and immunotherapy response. Ultimately, our findings demonstrated that ASRS, an age and senescence-related scoring system, was a robust predictor not only of LUAD patient prognosis but also of immunotherapy efficacy.

## Materials and methods

### Data collection

In this study, we analyzed 22 independent LUAD cohorts, of which only the TCGA-LUAD cohort was an RNA-seq dataset obtained from the USCS Xena website. The other 21 LUAD cohorts were Microarray datasets retrieved from the GEO database, including GSE68571, GSE3141, GSE11969, GSE68465, GSE8894, GSE13213, GSE11117, GSE19188, GSE14814, GSE29013, GSE31210, GSE26939, GSE29016, GSE37745, GSE42127, GSE30219, GSE50081, GSE63459, GSE72094, and GSE31546. We excluded samples without survival status, those with overall survival (OS) times <30 days, and duplicates, resulting in the inclusion of 3243 LUAD patients. We retrieved a total of 667 aging and senescence-related genes (ASRGs) from the Molecular Signatures Database (http://www.gsea-msigdb.org/), which are listed in Supplementary Table S1.

### Gene set variation analysis (GSVA)

In this study, we performed GSVA enrichment analysis of 667 ASRGs in 22 datasets, and quantified the activation level of aging and senescence-related pathway in each LUAD sample for subsequent analysis. Gene Set Variation Analysis (GSVA) is a non-parametric and unsupervised analytical approach that identifies enriched metabolic pathways in different samples by transforming the gene expression matrix of each sample into a gene set expression matrix. GSVA does not require prior differential analysis between samples as it can directly calculate the variation fraction of specific gene sets in each sample using the expression matrix.The following was the parameter settings: pvalueCutoff = 0.05, and pAdjustMethod = BH.

### Immune analysis

The expression profiles of different cohorts were entered in the “IOBR” package to obtain the final immune cell content matrix. In addition, we performed Spearman correlation analysis between ASRS and mRNA expression of immunomodulators. GSVA was also done with the same settings in anti-PD-1, anti-PD-L1, and anti-CTLA-4 immunotherapy groups, and survival and immune response were assessed.

### Drug prediction analysis

We investigated potential therapeutic drugs in the Genomics of Drug Sensitivity in Cancer (GDSC) and PRISM databases using ASRS.

### Statistical analysis

For continuous variables with a normal distribution and homogeneity of variance, a *t*-test with an independent sample was utilized. For non-normal distribution parameters, the Wilcoxon rank-sum test was performed. Via the Pearson correlation coefficient test, the correlation was analyzed.*p* values less than 0.05 were deemed statistically significant (*p < 0.05, **p < 0.01, and ***p < 0.001).

## Results

### ASRS based on GSVA in the multicenter study

**FIGURE 1:**
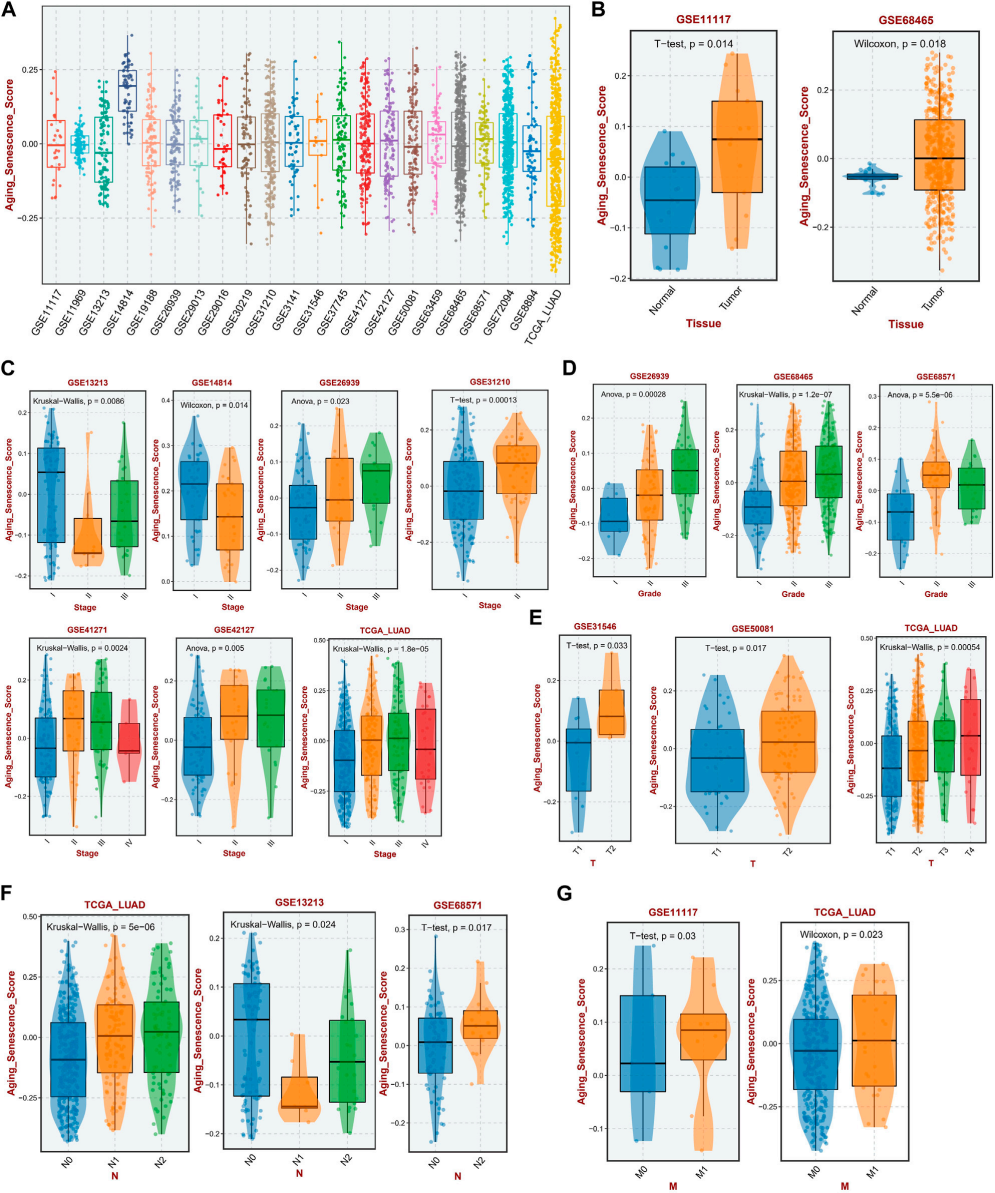
Relationship between ASRS and clinicopathological features in different cohorts. **(A)** The expression levels of ASRS in 22 independent cohorts. **(B)** Expression levels of ASRS in adjacent non-tumor and LUAD tissues in the GSE11117 and GSE68465 cohorts. Relationship between ASRS and different LUAD stage **(C)**, grade **(D)**, T-stage **(E)**, N-stage **(F)**, and M-stage **(G)** in different cohorts.

First, we conducted a GSVA analysis on all sample of 22 independent LUAD cohorts, in which the mean ASRS values were relatively close in most of the datasets, indicating that the ASRS levels were relatively close among LUAD patients in each independent cohort, and only the GSE14814 cohort was significantly outlier (Figure 1A). To further investigate whether ASRS is involved in disease progression in LUAD, we compared samples of different tissues, stages, and grades. In the GSE11117 and GSE68465 cohorts, the expression level of ASRS in tumor samples was considerably higher than that in neighboring normal tissues (Figure 1B). Stage II patients in the GSE13213 and GSE14814 cohorts had the lowest ASRS in tumor staging, and in the other cohorts ASRS increased with increasing staging. However, the ASRS of stage IV patients was lower in the GSE41271, GSE42127, and TCGA-LUAD cohorts, which may have been caused by the small number of stage IV patients, hence creating a substantial bias (Figure 1C). In terms of pathological grading, ASRS increased with increasing pathological grading in the GSE26939 and GSE68465 cohorts (Figure 1D). Similarly, in T-stage (Figure 1E), N-stage (Figure 1F), and M-stage (Figure 1G), ASRS significantly increased with disease progression. Taken together, changes in aging and senescence-related pathways in LUAD may be involved in disease progression.

### ASRS was associated with prognosis

**FIGURE 2:**
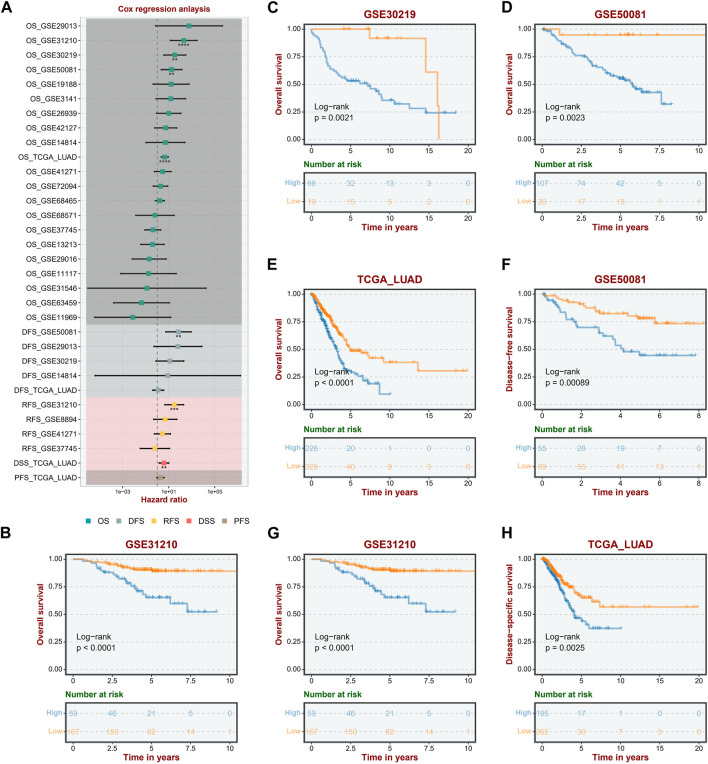
ASRS was associated with prognosis. **(A)** Correlation between ASRS expression and OS, DFS, RFS, DSS, and PFS of different cohorts. **(B–H)** Kaplan–Meier curves of OS, and DFS stratified by the low- and high-expression of ASRS in different cohorts.

To study further the correlation between ASRS and the prognosis of LUAD patients, the univariate Cox regression analysis and Kaplan-Meier survival analysis were performed firstly in the different cohorts and different survival outcomes. In the OS outcome, ASRS could serve as a prognostic risk factor for LUAD patients in the GSE31210, GSE30219, GSE50081 and TCGA-LUAD cohorts; in the DFS outcome, ASRS can be a risk factor for patients with LUAD in the GSE50081 cohort; in the RFS outcome, ASRS can be a risk factor for patients with LUAD in the GSE31210 and TCGA-LUAD cohorts (Figure 2A). In Kaplan-Meier survival analysis, with OS as the dependent variable, the high ASRS group had shorter survival times in the GSE31210 (Figure 2B), GSE30219 (Figure 2C), GSE50081 (Figure 2D), and TCGA-LUAD (Figure 2E) cohorts. In addition, among other survival outcomes, the high ASRS group was also an indicator of poor prognosis (Figures 2F–H).

### ASRS represents different immune pattern in TME

**FIGURE 3:**
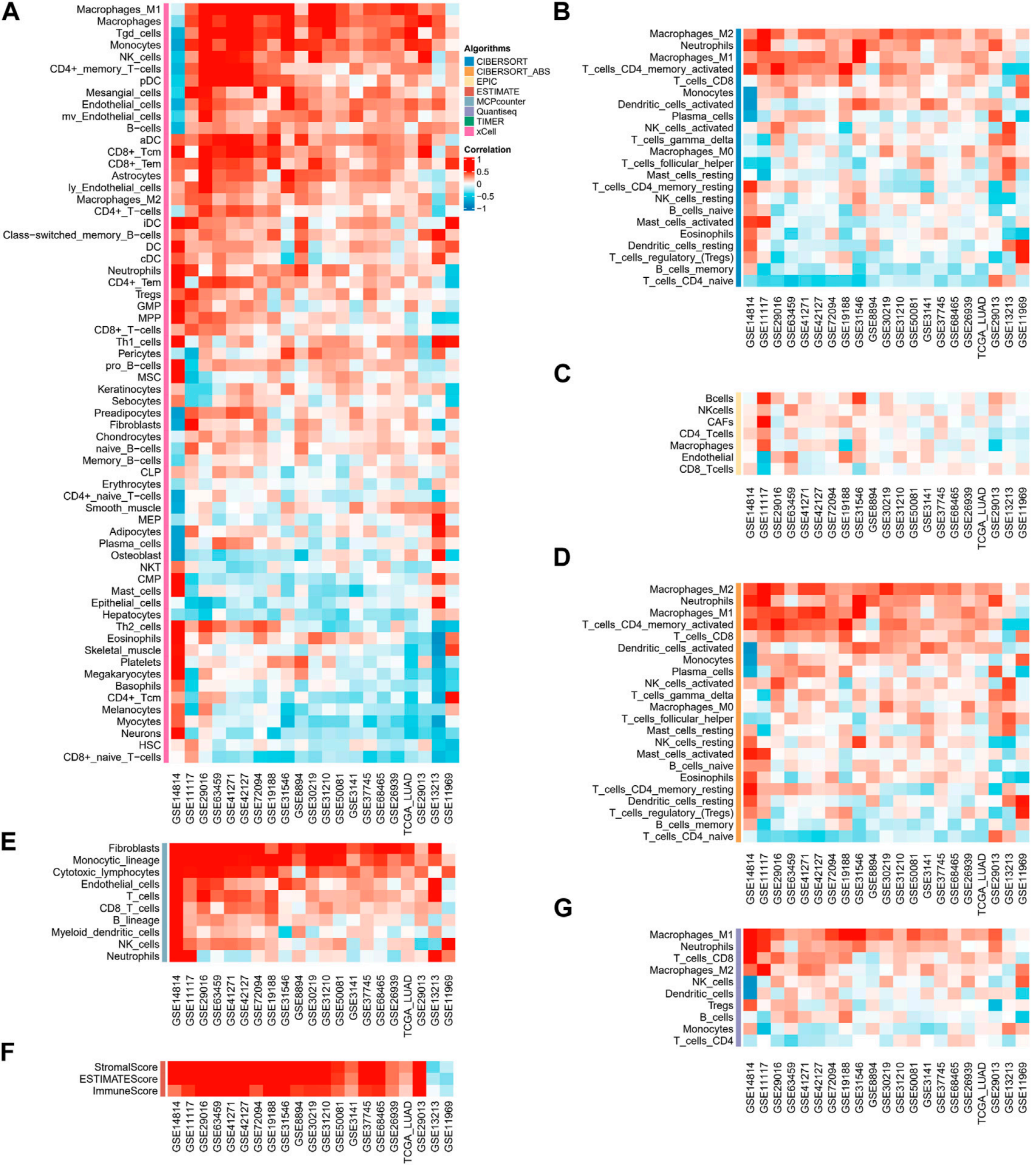
ASRS represents different immune pattern in TME. Correlation analysis between ASRS and different immune cells estimated by xCELL **(A)**, CIBERSORT **(B)**, EPIC **(C)**, CIBERSORT-ABS **(D)**, MCP-counter **(E)**, ESTIMATE **(F)**, and QUANTISEQ **(G)**.

In immune cell analysis, we estimated the abundance of immune cells in different samples using algorithms such as CIBERSORT-ABS, CIBERSORT, QUANTISEQ, MCP-counter, xCELL, EPIC, and ESTIMATE. In the majority of data sets, the xCELL algorithm revealed that ASRS was positively connected with Macrophages _ M2, Neutrophils, Macrophages_ M1, T _ cells _ CD4 _ memory _ activated and negatively correlated with T _ cells _ CD4 _naïve (Figure 3A). The CIBERSORT (Figure 3B) and EPIC algorithm (Figure 3C) showed that ASRS in most data sets was positively correlated with immune killer cells, such as CD8 _ T cells. In the CIBERSORT_ABS algorithm (Figure 3D), the results were similar to the CIBERSORT algorithm, and ASRS was positively correlated with Macrophages_M2, Neutrophils, Macrophages_M1, T_cells_CD^+^4_memory_activated, T_cells_CD^+^8, and Dendritic_cells_activated. The MCPcounter algorithm (Figure 3E) also showed the positive correlation between ASRS and Macrophages and CD^+^8 _ T cells. In addition, the ESTIMATE (Figure 3F) and QUANTISEQ (Figure 3G) algorithms also showed the strong indicating ability of ASRS in immune activation.

### ASRS was associated with immunomodulator-related mRNA expression

**FIGURE 4:**
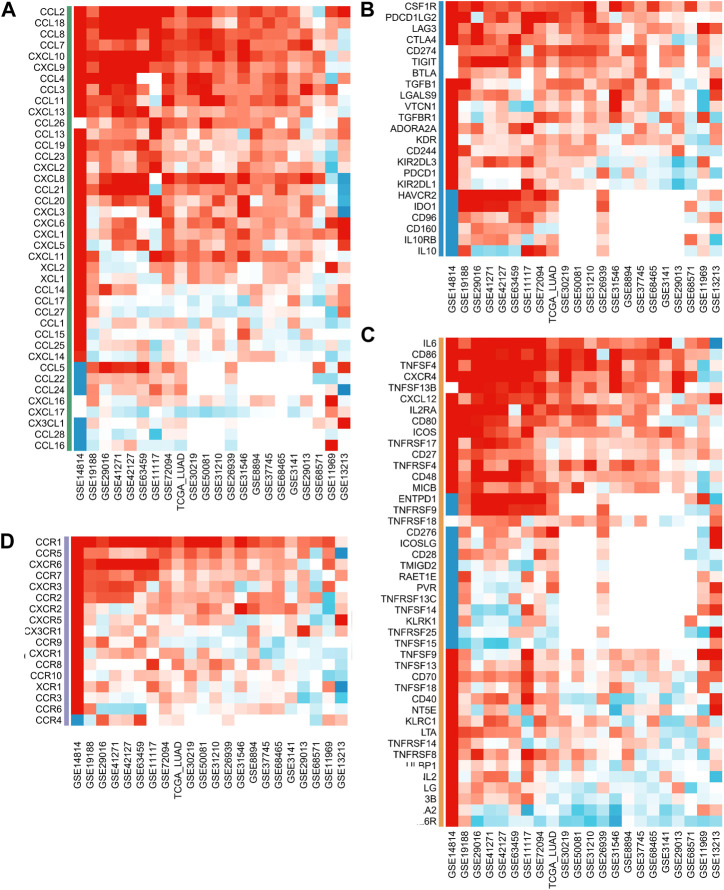
ASRS was associated with immunomodulator-related mRNA expression. Correlation analysis between ASRS and different immunomodulators estimated by chemokine **(A)**, immunoinhibitor **(B)**, immunostimulator **(C)**, and receptor **(D)** analysis.

We analyzed the correlation between ASRS and immunomodulators in each cohort. Chemokine analysis showed that ASRS was positively correlated with CCL2, CCL18, CCL8, CCL7, CXCL10, CXCL9, CCL4, CCL3, CCL11, CXCL13 and CCL26 in most cohorts (Figure 4A). Immunoinhibitor analysis showed that ASRS was positively correlated with CSF1R, PDCD1LG2, LAG3, CTLA4 and CD274 in most cohorts (Figure 4B). Immunostimulator analysis showed that in most cohorts, ASRS was negatively correlated with IL6R, HHLA2 and TNFRSF13B, but positively correlated with IL6, CD86, TNFSF4 and CXCR4 (Figure 4C). Finally, in receptor, ASRS in most cohorts was similarly positively correlated with CCR1, CCR5, CXCR6, etc. (Figure 4D).

### ASRS reflects immunotherapy response

**FIGURE 5:**
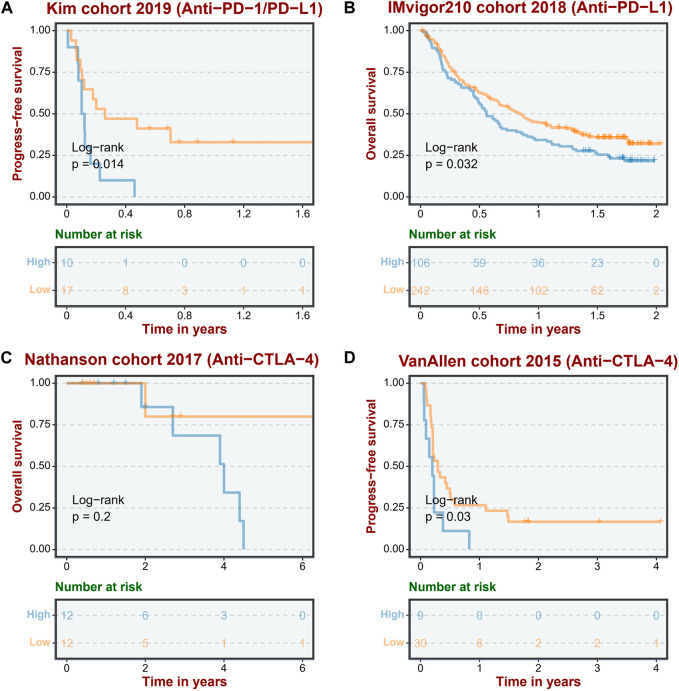
ASRS reflects immunotherapy response. The low ASRS is associated with a better response to immunotherapies in an anti-PD-L/PD-L1 cohort (Kim cohort 2019, **(A)**, an anti-PD-L1 cohort (IMvigor210 cohort 2018, **(B)**, and two anti-CTLA-4 cohorts (Nathanson cohort 2017, **(C)**; VanAllen cohort 2015, **(D)**.

Considering that ASRS can reflect the immunemodulator and immune infiltration landscape, we next sought to determine whether ASRS could predict patients’ responses to immunotherapy in the immunotherapy cohort. In Anti-PD-1 cohort, patients with high ASRS had shorter survival time (Figure 5A). Anti-PD-L1 cohort has the same prognostic indicator effect (Figure 5B). In addition, ASRS also has the same risk stratification ability in anti-CTLA-4 cohort (Figures 5C,D).

### ASRS could guide drug selection in LUAD patients

**FIGURE 6:**
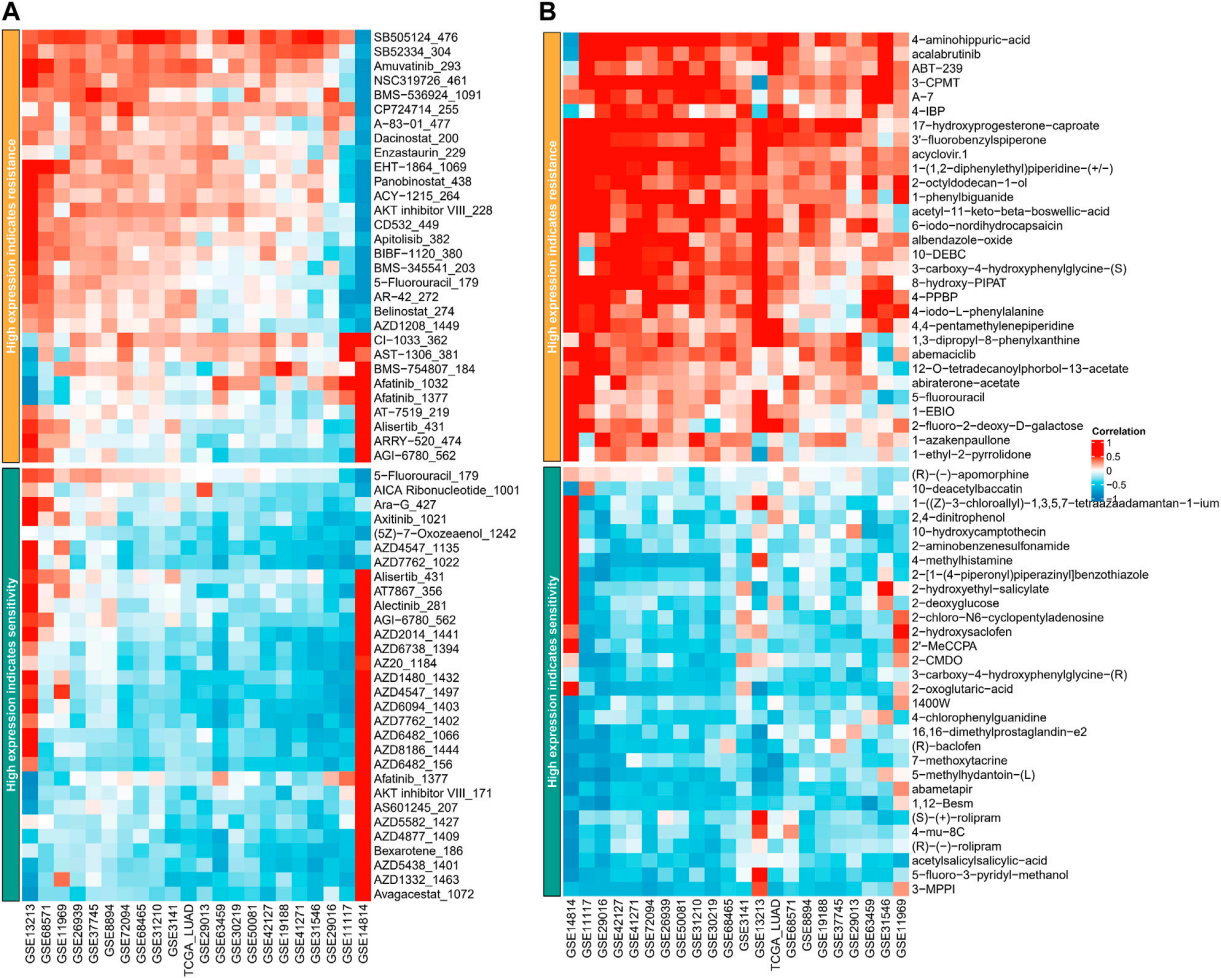
ASRS could guide drug selection in LUAD patients. Correlation analysis between ASRS and different drugs estimated in GDSC database **(A)** and PRISM database **(B)**.

The GDSC database analysis demonstrated that high ASRS in each cohort may indicate resistance to various drugs, such as SB505124 _ 476, SB52334 _ 304, Amuvatinib _ 293, NSC319726 _ 461, BMS-536924 _ 1091, CP724714 _ 255, A-83-01 _ 477, and Dacinostat _ 200, while being sensitive to other drugs including 5-Fluorouracil_179, AICA Ribonucleotide_1001, Ara-G_427, and Axitinib_1021, as shown in Figure 6A. Similarly, the PRISM database analysis revealed that high ASRS in each cohort may indicate resistance to certain drugs, such as 4−aminohippuric−acid, acalabrutinib, ABT−239, and 3−CPMT, while being sensitive to other drugs such as (R)−(−)−apomorphine, 10−deacetylbaccatin, and 1−(Z)−3−chloroallyl)−1, 3, 5, 7−tetraazaadamantan−1−ium, as shown in Figure 6B.

### ASRS reflects different gene alteration status

**FIGURE 7:**
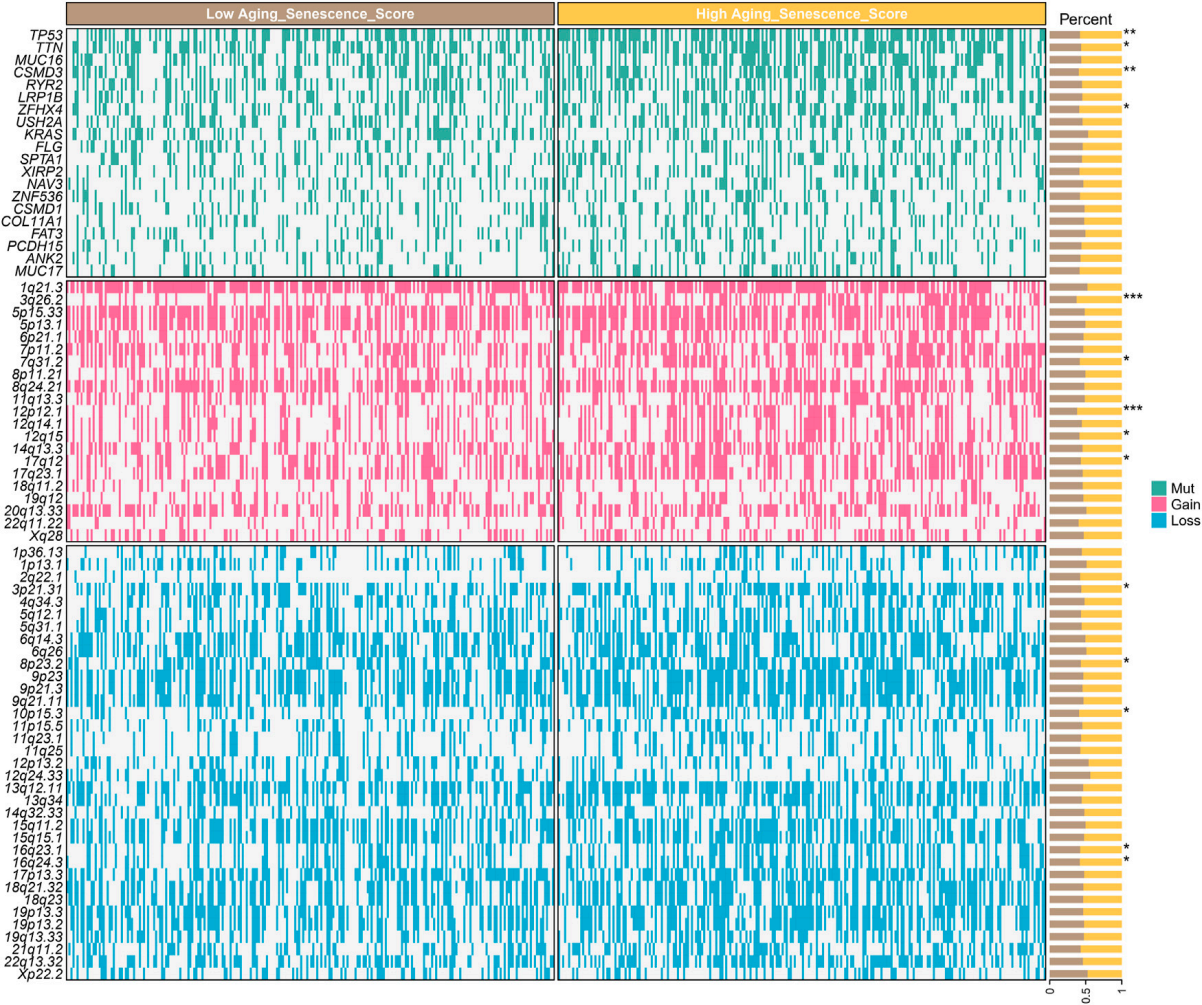
ASRS reflects different gene alteration status.

To further explore the reasons why different ASRS patients have different prognosis and drug response, we analyzed genomic differences among different ASRS patients in TCGA-LUAD cohort. Somatic mutations showed that TP53, TTN, CSMD3 and LRP1B were significant differences in patients with different ASRS. SNV amplification showed that 3q26.2, 7q31.2, 11q13.3, 12q14.1, and 14q13.3 were significant differences in patients with different ASRS. SNV deletion showed that 3p21.31, 8p23.2, 10p15.3, 16q23.1, 16q24.3 were significant differences in patients with different ASRS (Figure 7).

## Discussion

The field of oncology gained tremendous momentum in the 2000s as Hanahan and Weinberg distilled the complexity of cancer as a disease into six key features in their groundbreaking review of “hallmarks of cancer” (Hanahan and Weinberg, 2000). The hallmarks of cancer has recently been expanded to twelve, with cellular senescence being one of the latest findings (Hanahan, 2022). Cellular senescence is driven by multiple mechanisms, such as replication failure due to telomere shortening, oncogene activation, genotoxicity, nutrition and oxidative stress. Therefore, it is imperative to elaborate the impacts of the senescence on tumors for better cancer prevention and therapy. In fact, despite the fact that preclinical studies including cellular experiments and animal models have indicated broad effects of senescence on cancer (Mathon and Lloyd, 2001; Jochems et al., 2021; Liu et al., 2021), it is unknown whether senescent features of LUAD patients may be employed as biomarkers to guide clinical prognosis and treatment.

In this study, we established the senescence scoring to evaluate the senescence level of tumor tissues, termed as ASRS. Integrated analysis indicated a substantial correlation between ASRS and the prognosis and pathological characteristics of patients. Moreover, ASRS can function as a predictive indicator in a significant majority of LUAD cohorts. In the GSE11117 and GSE68465 cohorts, for instance, the expression level of ASRS in tumor samples was considerably higher than in neighboring normal tissues. Stage II patients in the GSE13213 and GSE14814 cohorts had the lowest ASRS in tumor staging, and in the other cohorts ASRS increased with increasing staging. In terms of pathological grading, ASRS increased with increasing pathological grading in the GSE26939 and GSE68465 cohorts. Similarly, in T-stage, N-stage, and M-stage, ASRS significantly increased with disease progression. In the OS outcome, ASRS could be a prognostic risk factor for LUAD patients in the GSE31210, GSE30219, GSE50081, and TCGA-LUAD cohorts; in the DFS outcome, ASRS can be a risk factor for LUAD patients in the GSE50081 cohort; and in the RFS outcome, ASRS can be a risk factor for LUAD patients in the GSE31210 and TCGA-LUAD cohort. The high ASRS group had shorter survival times in the GSE31210, GSE30219, GSE50081, and TCGA-LUAD cohorts. In addition, among other survival outcomes, the high ASRS group was also an indicator of poor prognosis.

Recent research has demonstrated that cellular senescence profoundly modifies the TME by promoting the aggregation of numerous types of immunosuppressive cells (Lasry and Ben-Neriah, 2015), which has far-reaching implications on the TME and tumor progression (Eggert et al., 2016). In particular, cellular senescence can change the fitness of immune cells and, eventually, influence the efficacy of cancer treatments, particularly immunotherapy (Choi et al., 2021). However, the associations between cellular senescence and TME remain unclear, and the utility of cellular senescence-related genes in assessing the immune infiltration of tumors requires additional study. In this work, the abundance of immune cells in different samples was estimated using different algorithms. We found ASRS as a characteristic of LUAD linked with an unique TME and immunological profile. Consequently, the discovery of the ASRS offers a new perspective on malignancies.

Furthermore, the ASRS is associated with different immunomodulator, immunotherapy response, drug selection, and genomic alterations. In the majority of cohorts, immunoinhibitor analysis revealed that ASRS was positively correlated with CSF1R, PDCD1LG2, LAG3, CTLA4, and CD274. Immunostimulator analysis showed that in most cohorts, ASRS was negatively correlated with IL6R, HHLA2 and TNFRSF13B, but positively correlated with IL6, CD86, TNFSF4 and CXCR4. Several studies have demonstrated that patients with low-senescore are more inclined to dramatic responses to immunotherapy such as ICIs (Lin et al., 2021a; Lin et al., 2021b). We next sought to determine whether ASRS could predict patients’ responses to immunotherapy in the immunotherapy cohort. In Anti-PD-1 cohort, patients with high ASRS had shorter survival time. Anti-PD-L1 cohort has the same prognostic indicator effect. In addition, ASRS also has the same risk stratification ability in anti-CTLA-4 cohort. Consistent with our previous data, it was shown that patients with a low ASRS were more likely to benefit from immunotherapy. More importantly, ASRS could guide drug selection in LUAD patients.

Despite the promising results, several issues need to be addressed. Firstly, it should be noted that the retrospective generation of the ASRS using publicly available databases may have introduced inherent selection bias. To establish the robustness and generalizability of the findings, large-scale prospective and multicenter clinical studies are essential. Additionally, it should be noted that certain critical clinical variables, such as chemoradiotherapy and surgery, were unavailable for analysis in some of the datasets, potentially affecting the accuracy of aging and senescence state analyses. To validate the expression of ASRS, a larger number of clinical pathology samples are required. Furthermore, to fully elucidate the roles of ASRS in the context of the disease, further *in vivo* and *in vitro* experiments are imperative.

## Conclusion

In conclusion, this is the first thorough analysis of aging and senescence in LUAD, leading to the identification of ASRS that are strongly linked with distinct TME and survival outcomes. Our research revealed that the ASRS can be used to identify not only individuals with varying OS and RFS risk levels, but also patients who would benefit from immunotherapy.

## Data Availability

The datasets presented in this study can be found in online repositories. The names of the repository/repositories and accession number(s) can be found in the article/Supplementary Material.

## Ethis statement

Ethical review and approval were waived for this study, due to the use of open-accessed data.
